# Vanillic Acid Modulates Antioxidant Defense and Methylglyoxal Detoxification Systems to Combat Drought Stress in Tomato Seedlings

**DOI:** 10.3390/plants13223114

**Published:** 2024-11-05

**Authors:** Khursheda Parvin, Mirza Hasanuzzaman, Sayed Mohammad Mohsin, Kamrun Nahar, Masayuki Fujita

**Affiliations:** 1 Department of Horticulture, Faculty of Agriculture, Sher-e-Bangla Agricultural University, Dhaka 1207, Bangladesh; 2 Department of Agronomy, Faculty of Agriculture, Sher-e-Bangla Agricultural University, Dhaka 1207, Bangladesh; 3 Department of Plant Pathology, Faculty of Agriculture, Sher-e-Bangla Agricultural University, Dhaka 1207, Bangladesh; 4 Department of Agricultural Botany, Faculty of Agriculture, Sher-e-Bangla Agricultural University, Dhaka 1207, Bangladesh; knahar84@yahoo.com; 5 Laboratory of Plant Stress Responses, Faculty of Agriculture, Kagawa University, Miki-cho, Kita-gun, Kagawa 761-0795, Japan

**Keywords:** water deficit stress, cellular damage, antioxidant defense, phenolic compound, glyoxalase enzyme, vegetable crops

## Abstract

Vanillic acid (VA) regulates various plant physiological and biochemical processes upon different environmental stresses to enhance their tolerance. This study aimed to evaluate the protective effect of VA on growth and physiology, including osmoprotection, and antioxidant defense systems for enhancing higher tolerance by lowering oxidative damage against water deficit stress in tomatoes (*Solanum lycopersicum* L. cv. BARI Tomato-16). Hydroponically grown tomato seedlings (8 d old) were pretreated with 50 µM VA for 2 days followed by water deficit stress (imposed by water withdrawal and 12% polyethylene glycol; PEG-6000) for 4 d. Drought stress inhibited the seedlings’ growth by reducing water content and photosynthetic pigments contents, alleviating oxidative stress induced by a reactive oxygen species and methylglyoxal. A significant enhancement in growth, biomass accumulation, and photosynthetic pigment content was observed in VA-pretreated stress conditions. In addition, there was an improvement in the water status and proline content, along with modulated activities of the antioxidant responses, including both non-enzymatic and enzymatic components in leaves of VA-pretreated seedlings upon the water deficit. Vanillic acid significantly reduced the reactive oxygen species generation and decreased cellular membrane damage in drought-affected tomato seedlings. Methylglyoxal detoxification was ensured to a great extent in VA-pretreated stressed tomato seedlings by strengthening the glyoxalase enzymes’ activities. Therefore, VA can be effective for protecting tomato seedlings by inducing a plant antioxidant defense and the methylglyoxal detoxification system and osmoregulation under drought stress.

## 1. Introduction

Sufficient water is one of the prime requirements for plant growth and survival. Therefore, water deficiency is a major reason for the restriction of plant growth, development, and yield [1,2]. Drought-induced considerable damages occur, which are understood as an alteration in morpho-physiological and biochemical levels, such as a modification in the leaf water status, inhibition in stomatal conductance and nutrient uptake, destruction in leaf pigments, and photosynthesis [3,4]. In addition, drought stress causes the elevated generation of reactive oxygen species (ROS) and consequent oxidative damage through cellular injury, including the peroxidation of lipids, oxidation of proteins, and breakdown of nucleic acid structures [1,5]. In opposition, plants are naturally equipped with enzymatic and non-enzymatic antioxidant components in an antioxidant defense system for regulating ROS and keeping those at a safer level [6,7]. The notable members of non-enzymatic antioxidants are phenolic compounds, tocopherol, ascorbic acid (AsA), and glutathione (GSH), while superoxide dismutase (SOD), catalase (CAT), glutathione *S*-transferase (GST), glutathione peroxidase (GPX), ascorbate peroxidase (APX), monodehydroascorbate reductase (MDHAR), dehydroascorbate reductase (DHAR), and glutathione reductase (GR) are antioxidant enzymes that work concurrently to reduce stress-mediated ROS generation [7].

In addition, osmoregulation is also likely to increase plant tolerance to drought [8]. Osmolytes like proline (Pro), glycine betaine, soluble sugar, and amine are efficient compatible solutes. Besides osmoregulation, they showed their roles in regulating ROS scavenging and stress signaling in the maintenance of cellular pH, in the soothing of cell membranes, and for various ultrastructural organelles, in making the cytosolic environment suitable for biochemical reactions [9,10]. Upregulation of the glyoxalase system is required to detoxify the excess methylglyoxal (MG) generation upon water-deficient conditions and help plants increase tolerance [8]. Glyoxalase enzymes like glyoxalase I (Gly I) and glyoxalase II (Gly II) work together and, thus, reduce MG content as well as cytotoxicity [11,12].

Beyond a certain level of stress, the plant lost its harmony in these defense tactics to tolerate deleterious effects that also vary with the plant species and ages. Therefore, these defensive mechanisms of plants demand additional support to reinforce the ROS detoxification under water deficit stress. Supplementation of phytohormone, plant nutrients, signaling molecules, organic acids, and antioxidants are thus becoming external efficient approaches for increasing plant stress tolerance by boosting up the plant’s inherent defense mechanisms. In search of new potential approaches, phenolic compounds have been receiving researchers’ attention for increasing plant stress tolerance due to their ability to scavenge ROS through structural functions and as secondary metabolites and antioxidants [13,14]. Vanillic acid (VA) is one kind of phenolic acid that has a robust antioxidant capacity because of its structural composition of hydroxyl groups and thus stabilizes phenoxyl radicals by providing hydrogen [15,16,17]. Stress tolerance of plants was regulated by exogenous VA, which have been reported by Quan and Xuan [18] and Xuan and Khang [19] in rice upon droughts and submergence, respectively, where they observed that growth parameters and photosynthetic pigment contents were increased upon VA application.

The role of VA in modulating osmolytes and increasing water uptake in drought-stressed plants was not studied. Vanillic-acid-induced regulation of glyoxalase and antioxidant defense system to reduce MG and ROS, respectively, were not investigated in drought-affected tomato plants. From our previous study [16,17], we were curious to apply VA to combat drought-induced suffering, including both physical and physiological water shortages in plants. Since VA works to strengthen the plant’s tolerance against abiotic stress as it is co-treated with salt and cadmium, what would happen if we used pretreated seedlings for upcoming stress? To implement the VA-induced plant stress tolerance in field conditions, we need to examine the precaution-based study. Therefore, due to these unclear matters from our previous studies, we conducted this present study to investigate the regulation of osmoregulation, antioxidant defense, and glyoxalase systems in tomato seedlings under water deficit condition through the exogenous VA application as a pretreatment basis. If we obtain a benefit from the VA-pretreatment approach to enhance plant stress tolerance, besides gaining a growth regulatory effect, then this ecofriendly and low-cost approach of higher tomato production technology could be more effective in practical farming. Further questions, such as how VA reduces oxidative damage to the membrane and the photosynthetic pigments and how it improves biomembrane properties, phenotypic appearances, and the growth performance of tomato seedlings, were also examined in the present study. Exploring the nature of VA being effective for obtaining two-way benefits, including being both a growth promoter and an influencer for stress tolerance against drought for tomatoes, provides us with new insights about the role of VA in this study.

## 2. Materials and Methods

### 2.1. Seedling Establishment and Treatment

Healthy and uniform size of tomato seeds (*Solanum lycopersicum* cv. BARI Tomato-16) were sown on Petri plates containing two layers of filter papers. After 5 days, 40 germinated seedlings were kept in each plate, and the plates were transferred to growth chambers under a controlled environment (temperature, light, photoperiod, and relative humidity were 25 ± 2 °C, 350 μmol photon m^−2^ s^−1^, 16/8 h of light/dark, and 65–70%, respectively). Seedlings were grown in full-strength Hoagland nutrient solution [20]. The 8 d old seedlings containing 18 Petri plates were divided into two sets, where one set was pretreated with VA (50 µM; co-applied with nutrient solution) for two days, and another set was grown with only a nutrient solution. After that, seedlings were subjected to drought stress (12% PEG-polyethylene glycol and water deficit) for 4 days. A control treatment was applied with the nutrient solution only. Treatment combinations were a control (without any stress and VA), only VA pretreatment, W0 (withdrawal of water), VA-pretreated subjected to withdrawal of water, PEG (PEG-treated only), and VA-pretreated subjected to PEG stress. After four days of drought exposure, seedlings were considered for various data collection, where leaves of the third and fourth positions were collected to measure biochemical data. This whole study consisted of three different experiments that were conducted at repeated times, including three replications for each treatment. The experimental design was a completely randomized block design.

### 2.2. Growth Measurement and Biomass Accumulation

The length of shoots and roots, their fresh weight (FW), and dry weight (DW) were measured from ten seedlings from each treatment. The FW was taken from harvested samples, while the DW was taken after drying the samples at 80 °C for 72 h.

### 2.3. Photosynthetic Pigment Determination

Fresh leaves were extracted with absolute ethanol (1 h heated in a hot bath at 60 °C), and after being cooled, the samples were used to measure the content of chlorophyll (Chl *a* and *b*) and carotenoid (Car) following the protocol of Wellburn [21]. Chl *a*, Chl *b*, and Car were calculated from the spectrophotometric values taken at 664, 648, and 470 nm, respectively.

### 2.4. Relative Water Content and Proline Accumulation

The leaf RWC was measured according to Barrs and Weatherley [22] from the FW, turgid weight (TW), and DW of leaf samples. The following equation was used to calculate the leaf RWC:

RWC (%) = (FW − DW)/(TW − DW) × 100

Proline content was measured according to Bates et al. [23]. In brief, we extracted supernatant from fresh leaves by sulfo-salicylic acid (3%); then, the supernatant was mixed with glacial acetic acid and acid ninhydrin, and after that, these were heated at 100 °C for 1 h. Then, after being cooled, toluene was added to separate the ninhydrin-proline complex and read spectrophotometrically at 520 nm to determine the Pro content. A standard curve of known concentration of Pro was used to compare and determine the Pro content leaves.

### 2.5. Histochemical Detection of ROS

Leaves were subjected to histochemical staining with nitro blue tetrazolium (NBT) and 3,3-diaminobenzidine (DAB) to detect the spots of O_2_^•^⁻ and H_2_O_2_, respectively [24].

### 2.6. Measurement of H_2_O_2_, Lipid Peroxidation and Electrolyte Leakage

The content of H_2_O_2_ was measured from the supernatant obtained from extracted fresh leaves by 5% TCA [25], where potassium iodide (KI) and a potassium phosphate buffer (K–P; pH 7.0) were used to measure the absorbance at 390 nm spectrophotometrically and expressed as nmol g^−1^ FW.

An indicator of lipid peroxidation, malondialdehyde (MDA) content was measured according to the method of Heath and Packer [26], with slight modification from the method of Hasanuzzamant et al. [27] based on thiobarbituric acid (TBA) reactive substances. Thereafter, the measurement was obtained from the optical absorbance difference between 532 and 600 nm and calculated using an extinction coefficient of 155 mM^−1^ cm^−1^ expressed as nmol g^−1^ FW. Hence, the absorbance was measured spectrophotometrically at 532 nm and corrected at 600 nm.

Indication of cell membrane damage, electrolyte leakage (EL) was determined from the leaf and root tissues according to Dionisio-Sese and Tobita [28]. In detail, 0.2 g of fresh leaves were chopped into less than 1 cm size smaller pieces and immersed into 20 mL of DH_2_O containing a glass tube covered with a cap, which was later incubated at 35 °C for 1 h. First, electrical conductivity (EC, E1) was measured from that using a CON 700 electrical conductivity meter (Eutech Instruments, Singapore). After that, these tubes were subjected to another autoclave (121 °C) for 20 min, and a second EC (E2) was obtained after cooling. Finally, EL was obtained by using the following equation, EL (%) = E1/E2 × 100.

### 2.7. Assessment of Ascorbate and Glutathione Content

According to the method by Kampfenkel et al. [29], leaf extraction was performed using 5% TCA and then centrifuged at 11,500× *g* for 15 min. The collected supernatant was used to determine the total AsA, reduced form of AsA, total GSH, reduced form of GSH, and oxidized glutathione (GSSG), following the instructions described by Hasanuzzaman et al. [27]. The extracted supernatant was neutralized by using a 0.5 M K–P buffer (pH 7.0) and dithiothretitol (DTT, 0.1 M), where DH_2_O acted as a substrate for determining the total AsA and reduced AsA, respectively, at 265 nm spectrophotometrically, employing a standard curve of known concentration. Thereafter, dehydroascorbate (DHA) was calculated by deducting AsA from the total ascorbate.

In the case of the GSH pool, the extracted supernatants were neutralized by a K–P buffer (0.5 M, pH 7.0) and DH_2_O for the total GSH, while 2-vinylpyridine was used for GSSG instead of DH_2_O. Both total GSH and GSSG were measured by observing the absorbance at 412 nm spectrophotometrically based on enzymatic recycling using 5,5-dithio-bis (2-nitrobenzoic acid) (DTNB), GR, and nicotinamide adenine dinucleotide phosphate (NADPH). A previously prepared standard curve of known concentration of GSH was used to determine the total GSH and GSSG content, where the GSH content was calculated by deducting the GSSG from the total GSH.

### 2.8. Enzyme Extraction and Protein Quantification

Freshly harvested leaves (0.5 g) were extracted by a buffer solution (1 mL) containing a K–P buffer (50 mM, pH 7.0), potassium chloride (100 mM KCl), AsA (1 mM), β-mercaptoethanol (5 mM), and glycerol (10%, *w*/*v*) using a pre-cooled mortar and pestle. Then, extracted samples were centrifuged (11,500× *g* at 4 °C) for 12 min and obtained a distinct supernatant, which was used to measure the activities of the enzymes [17].

Protein estimation from the crude of the enzyme extraction was performed spectrophotometrically at 595 nm with the help of a standard curve of known concentration using bovine serum albumin (BSA) [30].

### 2.9. Assays of the Activities of Enzymes

The activity of CAT (EC: 1.11.1.6) was assayed following Hasanuzzaman et al. [27].

The reaction buffer (50 mM K–P buffer, pH 7.0, and 15 mM H_2_O_2_) and enzyme extract were mixed to take a spectrophotometer reading, where the decrease in absorbance was recorded for 1 min at 240 nm. The activity of CAT was later computed by using 39.4 M^−1^cm^−1^ as the extinction coefficient.

The GR activity (EC: 1.6.4.2) was measured from the reaction between the reaction mixture and enzyme/plant sample [27]. In brief, the reaction mixture contained 0.1 M K–P buffer (pH 7.0), 1 mM GSSG, 1 mM EDTA, and 0.2 mM NADPH. The reaction was initiated with GSSG, which was also NADPH-dependent, and the declining absorbance was monitored at 340 nm and later 6.2 mM^−1^ cm^−1^, as an extinction coefficient was used to calculate the actual GR activity.

Following the instruction of Hasanuzzaman et al. [27], GST activity (EC: 2.5.1.1) was quantified. The increasing trend in absorbance at 340 nm was read for 1 min from the reaction among enzymes, GSH and 1-chloro-2,4-dinitrobenzene (CDNB). The enzyme activity was then computed by using 9.6 mM^−1^cm^−1^ as the extinction coefficient.

The SOD (EC: 1.15.1.) activity was estimated following the method of El-Shabrawi et al. [31] and expressed as a U min^−1^ mg^−1^ protein, where U means the required enzyme for the 50% inhibition of NBT.

The activity of APX (EC: 1.11.1.1) was measured at 290 nm spectrophotometrically. We used an assay mixture with K–P buffer (50 mM; pH 7.0), EDTA (0.1 mM), AsA (0.5 mM), H_2_O_2_ (0.1 mM), and enzyme [32]. The extinction coefficient was 2.8 mM^−1^ cm^−1^ for computing the final activity.

The MDHAR (EC: 1.6.5.4) and DHAR (EC: 1.6.5.4) activities were determined as per the description of Nahar et al. [33], where the decreasing and increasing absorbance at 265 nm and 340 nm were observed, respectively. The MDHAR activity was assayed from the reaction mixture of NADPH (0.2 mM), Tris-HCl buffer (50 mM, pH 7.5), AsA (2.5 mM), ascorbate oxidase (AO; 0.5 units), and enzyme. The reactions from the mixture of the K–P buffer, DHA, EDTA, GSH, and the enzyme were observed for DHAR activity.

The GPX (EC: 1.11.1.9) activities were determined by following Nahar et al. [33] from the decreasing trends of absorbance at 340 nm from the reaction of enzymes with reaction mixture (K–P buffer, EDTA, GSH, NaN_3_, NADPH, GR, and H_2_O_2_).

The glyoxalase enzymes’ activity, like Gly I (EC: 4.4.1.5) and Gly II (EC: 3.1.2.6), was estimated by following Hasanuzzaman et al. [27] and Principato et al. [34], respectively. An increase in absorbance at 240 nm was observed for Gly I activity from the mixture of the K–P buffer, GSH, MgSO_4_, MG, and enzyme. On the other hand, for Gly II activity, the reaction mixture was prepared from the enzyme, Tris-HCl buffer, DTNB, and *S*-D-lactoylglutathione, and from that, the increasing absorbance at 412 nm was read.

### 2.10. Assey of Methylglyoxal Content

Perchloric acid (PCA) was used as an extraction buffer to measure the content of MG, according to Nahar et al. [33], followed by centrifugation at 11,000× *g*. The *N*-acetyl-L-cysteine was added to the collected supernatant to read spectrophotometrically at 288 nm, and finally, the MG content was calculated by using a standard curve of known concentration.

### 2.11. Statistical Analysis

The collected mean data from the three replications (n = 3) were subjected to statistical analysis using CoStat v.6.400 computer-based software (CoHort Software, Monterey, CA, USA) [35]. The one-way ANOVA technique was used to analyze the data, and Fisher’s least significant difference (LSD) test was used to compare the mean difference with 5% level of significance.

## 3. Results

### 3.1. Growth Measurement

**Figure 1:**
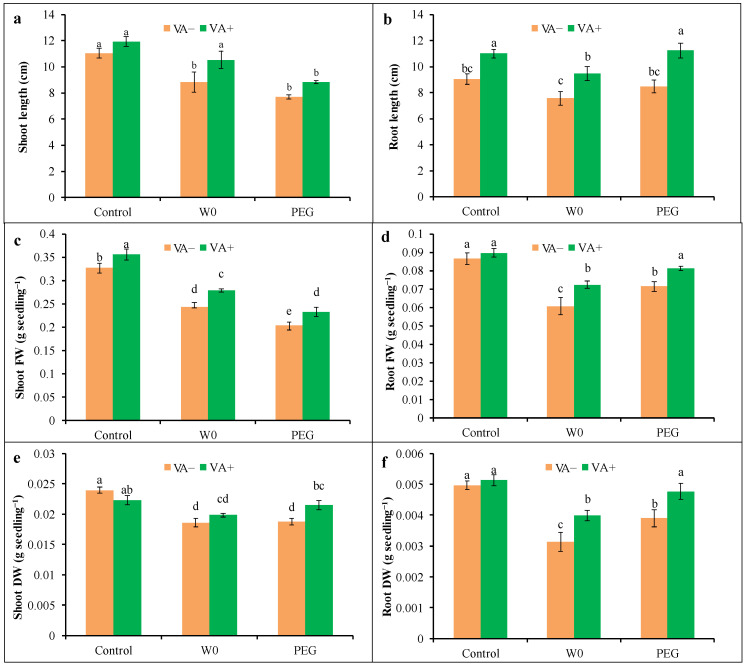
Modulation of growth ((**a**): shoot length; (**b**): root length) and fresh weight and dry weight ((**c**): shoot FW; (**d**): root FW; (**e**): shoot DW; (**f**): root DW) of tomato seedlings grown in hydroponic nutrient solution by non-pretreated and pretreated with VA (50 μM; 2 d) under drought stress (4 d). Here, C: control, VA: vanillic acid, W0: without water, PEG: 12% PEG. Mean value (±SD) was calculated from the three replications (n = 3) for each treatment; dissimilar letters in a column refer to significant different among the treatments at *p* ≤ 0.05 applying Fisher’s LSD test.

**Figure 2:**
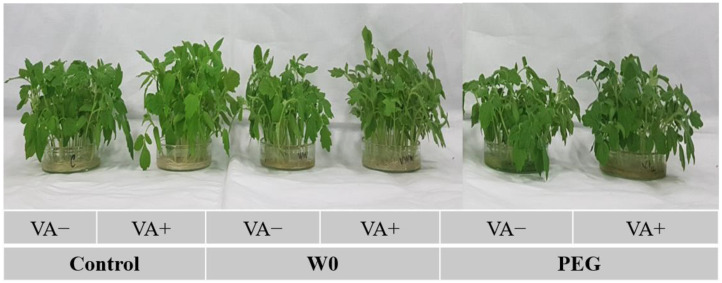
Phenotypic appearances of tomato seedlings grown in hydroponic nutrient solution by non-pretreated and pretreated with VA (50 μM; 2 d) under drought stress (4 d). Here, C: control, VA: vanillic acid, W0: without water, PEG: 12% PEG).

Tomato growth was hampered by both the water deficit and PEG-induced drought, as expressed by the reduction in the shoot length, root length, and DW and FW of the shoot and root (Figure 1). In brief, a water deficit and PEG-mediated stress caused the reduction in length of both the shoot and root (by 20 and 16%, respectively) and the FW of the shoot and root (26 and 30%, respectively) and their DW (22 and 37%, respectively), where it was measured that the maximum root growth retardation happened due to the water deficit, while PEG caused the highest shoot growth retardation. Thereafter, VA pretreatment resulted in the betterment of drought-induced growth inhibition, where significant elongation of both the shoot and root by 19 and 25%, respectively, was observed, along with an increased FW and DW of both the shoot and root in VA-pretreated seedlings under water deficit conditions. In the case of PEG-induced stress, VA pretreatment caused a significant improvement in root elongation by 33%, with biomass accumulation of both the shoot and root. Pictorial presentations of tomato seedlings’ growth under drought stress with and without VA pretreatment have been presented in Figure 2.

### 3.2. Osmotic Stress and Proline Accumulation

**Figure 3:**
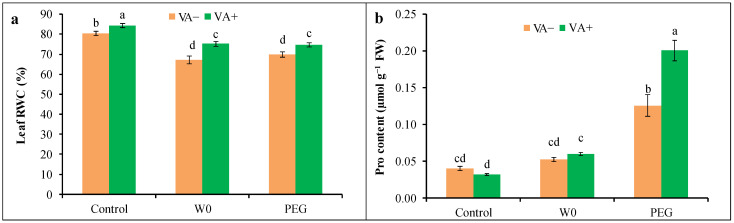
Water status ((**a**): Leaf RWC; (**b**): Pro content) of tomato seedlings grown in hydroponic nutrient solution by non-pretreated and pretreated with VA (50 μM; 2 d) under drought stress (4 d). Here, C: control, VA: vanillic acid, W0: without water, PEG: 12% PEG. Mean value (±SD) was calculated from the three replications (n = 3) for each treatment; dissimilar letters in a column refer to significant different among the treatments at *p* ≤ 0.05 applying Fisher’s LSD test.

A drought-induced significant reduction in the leaf RWC was measured in both water scarcity and PEG-treated seedlings by 16 and 13%, respectively, compared to the control, which indicates the suffering of the tomato from PEG stress (Figure 3a). Thereafter, Pro accumulation was elevated in stressed conditions by 215% under PEG conditions in comparison to without-stress conditions (Figure 3b). However, VA application caused the alteration in Pro as well as RWC, where further maximum Pro accumulation (about 60% higher than respective stress) in PEG-mediated stress conditions was measured (Figure 3).

### 3.3. Photosynthetic Pigment Accumulation

**Figure 4:**
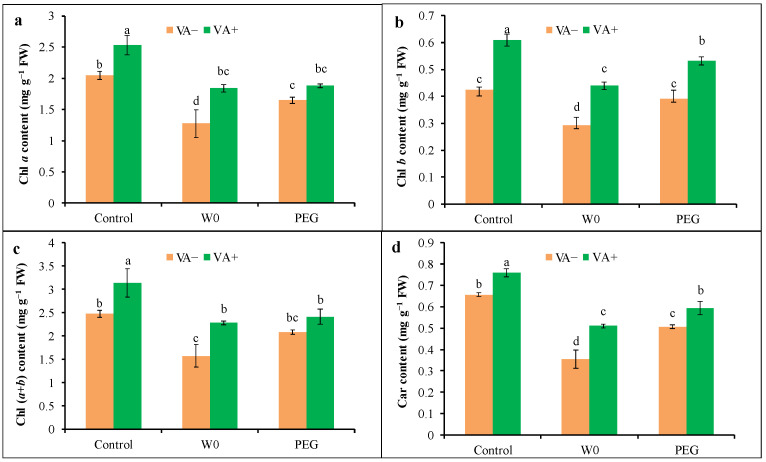
Chlorophyll content ((**a**): Chl *a*; (**b**): Chl *b*; (**c**): Chl *a*+*b*; (**d**): Car) of tomato seedlings grown in hydroponic nutrient solution by non-pretreated and pretreated with VA (50 μM; 2 d) under drought stress (4 d). Here, C: control, VA: vanillic acid, W0: without water, PEG: 12% PEG. Mean value (±SD) was calculated from the three replications (n = 3) for each treatment; dissimilar letters in a column refer to significant different among the treatments at *p* ≤ 0.05 applying Fisher’s LSD test.

A water shortage caused the reduction in photosynthetic pigments like Chl *a*, Chl *b*, Chl (*a*+*b*), and Car by 38, 31, 36, and 46%, respectively, while 19, 8, 16, and 23% were, respectively, by PEG treatment (Figure 4). Thus, water-scarcity-mediated drought stress was comparatively more prominent in the case of the destruction of Chl and Car content than PEG-mediated osmotic stress. In addition, VA pretreatment caused the recovery of both Chl and Car content regarding both the water deficit and osmotic stress. The VA-pretreated seedlings showed about 44, 50, 45, and 44% higher Chl *a*, Chl *b*, Chl (*a*+*b*), and Car content, respectively, under water deficit stress, while the increments in the case of Chl *b* and Car were significant in VA-treated PEG-stressed seedlings compared to respective stress alone (Figure 4).

### 3.4. Indication of Oxidative Stress

**Figure 5:**
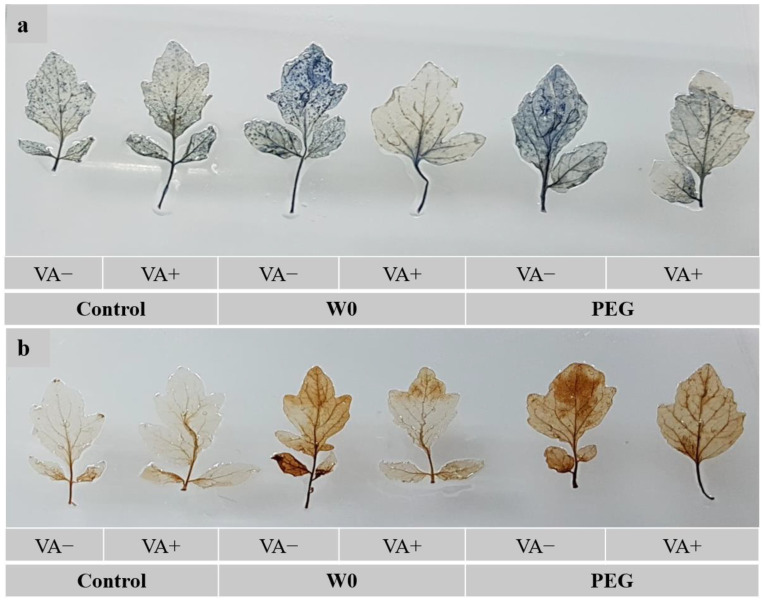
Histochemical detection of ROS accumulation ((**a**): O_2_^•−^; (**b**): H_2_O_2_) in leaves of tomato seedlings grown in hydroponic nutrient solution by non-pretreated and pretreated with VA (50 μM; 2 d) under drought stress (4 d). Here, C: control, VA: vanillic acid, W0: without water, PEG: 12% PEG).

Elevated ROS like O_2_^•^⁻ and H_2_O_2_-induced blue and deep brown spots, respectively, were evident in both water deficient and PEG-induced stressed leaves, compared to without stress-treated leaves. Conversely, VA-pretreated leaves showed a reduction in the generation of O_2_^•^⁻ and H_2_O_2_-mediated spots under water deficit and PEG-induced drought conditions compared to stress alone (Figure 5).

**Figure 6:**
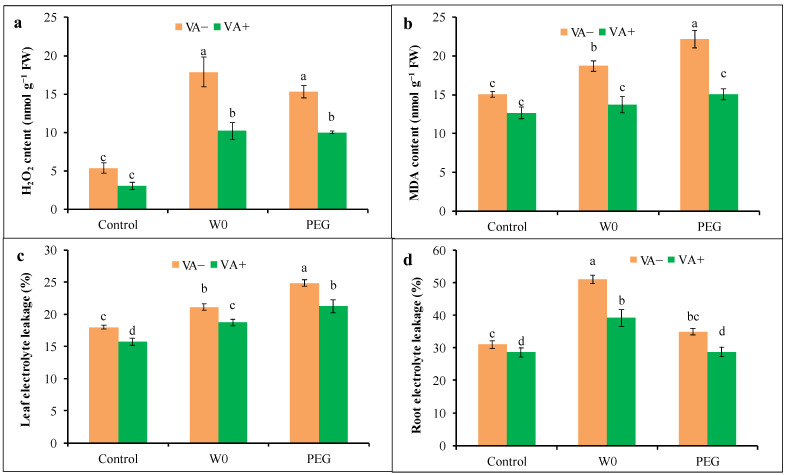
Oxidative stress markers ((**a**): H_2_O_2_ content; (**b**): MDA content; (**c**): Leaf electrolyte leakage; (**d**): root electrolyte leakage) of tomato seedlings grown in hydroponic nutrient solution by non-pretreated and pretreated with VA (50 μM; 2 d) under drought stress (4 d). Here, C: control, VA: vanillic acid, W0: without water, PEG: 12% PEG. Mean value (±SD) was calculated from the three replications (n = 3) for each treatment; dissimilar letters in a column refer to significant different among the treatments at *p* ≤ 0.05 applying Fisher’s LSD test.

The level of H_2_O_2_ was elevated by 235 and 187% in water withdrawal and PEG-induced drought-treated seedlings, respectively, compared to the control treatment, which is similar to the findings of histochemical detection. On the other hand, VA-pretreated seedlings showed a lower accumulation of H_2_O_2_ under both a water deficit and PEG stress in contrast to the respective stress alone (Figure 6a). The accumulation of MDA as an indicator of lipid peroxidation was elevated by 24 and 47% in tomatoes grown under W0 and PEG treatment, respectively, in contrast to the control (Figure 6b). However, VA-pretreated seedlings showed a reduction in the MDA content by 27 and 32% in W0 and PEG treatment, respectively, compared to their respective stress levels alone.

Drought-induced higher membrane damage was indicated by elevated EL in both leaves and root tissues of tomato seedlings under both water deficit and PEG treatment. Water scarcity caused the highest EL by 18 and 64% from leaves and roots, respectively, while in the case of a PEG-induced drought, the leaf EL reduction was significant (about 38%) compared to the control. However, VA-pretreated seedlings showed a reduction in EL from both leaves and root tissue under both water deficit and osmotic-induced drought stress compared to their respective stress alone (Figure 6c,d).

### 3.5. Antioxidant’s Defense System

#### 3.5.1. Enzymatic Antioxidant Activities

**Figure 7:**
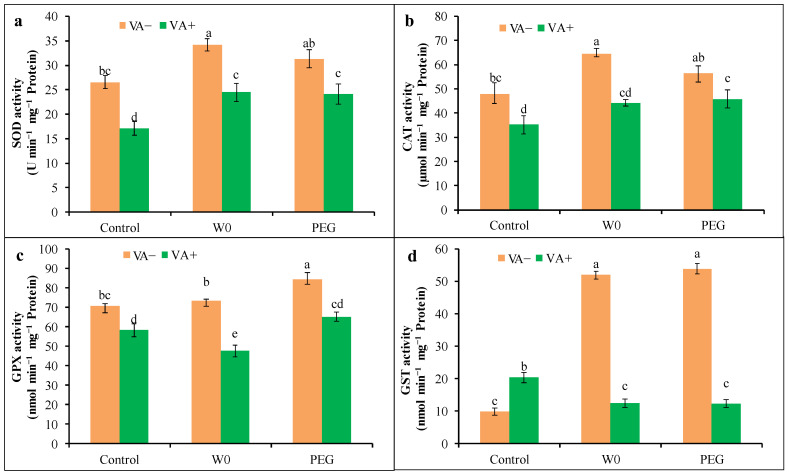
Activities of SOD (**a**), CAT (**b**), GPX (**c**), and GST (**d**) in leaves of tomato seedlings grown in hydroponic nutrient solution by non-pretreated and pretreated with VA (50 μM; 2 d) under drought stress (4 d). Here, C: control, VA: vanillic acid, W0: without water, PEG: 12% PEG. Mean value (±SD) was calculated from the three replications (n = 3) for each treatment; dissimilar letters in a column refer to significant different among the treatments at *p* ≤ 0.05 applying Fisher’s LSD test.

Drought stress increased the activity of SOD, CAT, GPX, and GST in tomato seedlings compared to the control (Figure 7). A water deficit caused the higher activity of SOD and CAT by 29 and 35%, respectively. The GPX activity significantly increased in PEG treatment compared to the control, while in the case of GST, a significant increment was recorded in both kinds of stresses (W0 and PEG; about 432 and 452%, respectively) compared to the control. However, VA-pretreated seedlings under drought stress had lower activity of these enzymes compared to respective stress alone (Figure 7). The VA pretreatment caused a significant reduction in SOD, CAT, GPX, and GST activities by 28, 31, 35, and 76%, respectively, in W0 and 23, 19, 23, and 77%, respectively in PEG stress conditions, than their respective stress alone.

**Figure 8:**
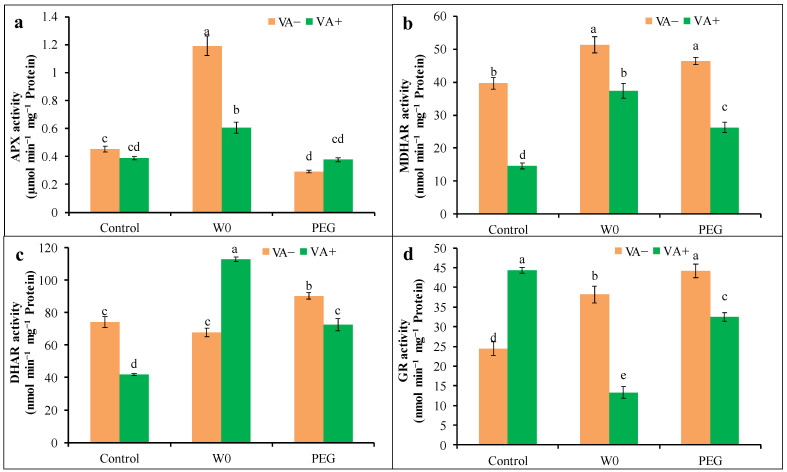
Activities of AsA-GSH pathway enzymes; APX (**a**), MDHAR (**b**), DHAR (**c**), and GR (**d**) in leaves of tomato seedlings grown in hydroponic nutrient solution by non-pretreated and pretreated with VA (50 μM; 2 d) under drought stress (4 d). Here, C: control, VA: vanillic acid, W0: without water, PEG: 12% PEG. Mean value (±SD) was calculated from the three replications (n = 3) for each treatment; dissimilar letters in a column refer to significant different among the treatments at *p* ≤ 0.05 applying Fisher’s LSD test.

The water shortage elevated the activity of APX by 165%, but the PEG-induced stress decreased this activity by 35% compared to the control. Conversely, VA pretreatment altered the stress-induced APX activity in tomato seedlings, which was reduced (49%) in water deficit conditions compared to respective stress alone (Figure 8a). In this study, MDHAR, DHAR, and GR activities were increased under PEG treatment conditions, while in W0 treatment conditions, DHAR activity was static to the control, along with a higher activity of MDHAR and GR. However, MDHAR, DHAR, and GR enzymatic activities were significantly reduced in VA-pretreated seedlings upon PEG-induced stress, while in the case of W0, VA pretreatment caused the increment in DHAR activity, along with lower activity from MDHAAR and GR compared to respective stress alone (Figure 8).

#### 3.5.2. Non-Enzymatic Antioxidant Levels

**Figure 9:**
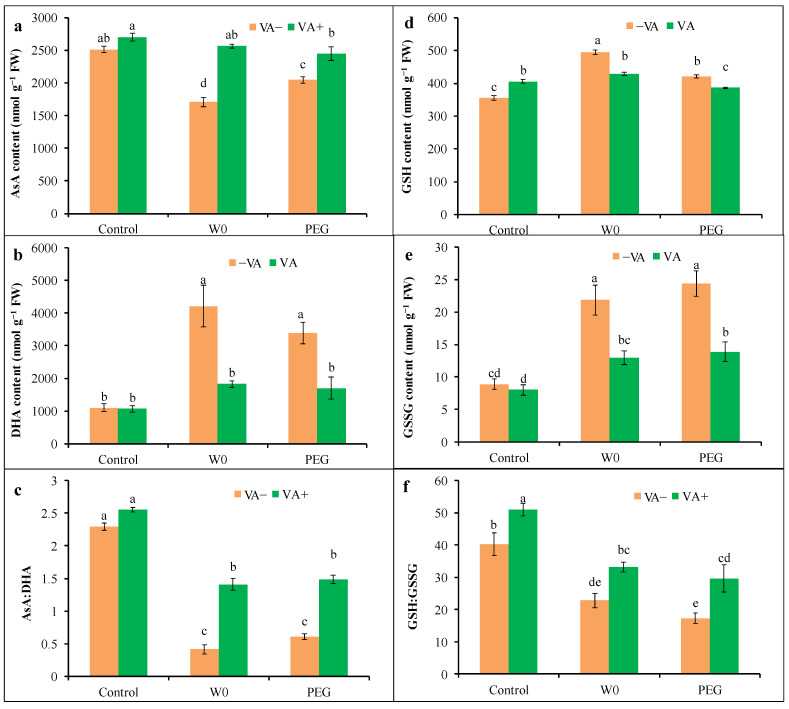
Content of AsA (**a**), DHA (**b**), GSH (**d**), and GSSG (**e**) and redox status of AsA:DHA (**c**) and GSH:GSSG (**f**) of tomato seedlings grown in hydroponic nutrient solution by non-pretreated and pretreated with VA (50 μM; 2 d) under drought stress (4 d). Here, C: control, VA: vanillic acid, W0: without water, PEG: 12% PEG. Mean value (±SD) was calculated from the three replications (n = 3) for each treatment; dissimilar letters in a column refer to significant different among the treatments at *p* ≤ 0.05 applying Fisher’s LSD test.

In comparison to the control, drought caused a decrease in AsA content with a higher content of DHA, which resulted in a reduction in AsA:DHA (Figure 9c). A water deficit caused a reduction in AsA:DHA by 82% because of a 32% reduction in AsA with higher DHA content (284%), whereas AsA:DHA reduced by 74% in the case of the PEG-induced drought. But VA pretreatment caused the alteration of AsA:DHA, which was elevated by 240 and 145% in W0- and PEG-treated conditions, respectively, compared to their respective stress alone because VA-pretreated seedlings showed an increase in AsA content with the lowering of DHA in drought stress conditions (Figure 9a,b). Moreover, both W0 and PEG treatment elevated the content of both GSH and GSSG, which resulted in a reduction in GSH:GSSG by 43 and 57%, respectively, compared to the control (Figure 9d–f). However, VA-pretreated seedlings had a lower content of both GSH and GSSG under both W0- and PEG-induced drought stress conditions. Thus, VA pretreatment enhanced GSH:GSSG by 45 and 71% in W0- and PEG-induced drought conditions, respectively, in comparison with respective stress alone.

### 3.6. Methylglyoxal Content and Glyoxalase Enzyme Activities

**Figure 10:**
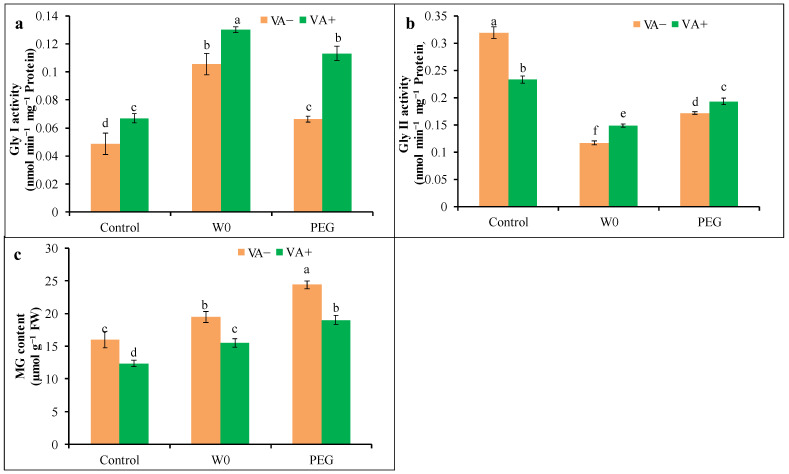
Activities of glyoxalase enzymes ((**a**): Gly I, (**b**): Gly II) and MG content (**c**) in leaves of tomato seedlings grown in hydroponic nutrient solution by non-pretreated and pretreated with (50 μM; 2 d) under drought stress (4 d). Here, C: control, VA: vanillic acid, W0: without water, PEG: 12% PEG. Mean value (±SD) was calculated from the three replications (n = 3) for each treatment; dissimilar letters in a column refer to significant different among the treatments at *p* ≤ 0.05 applying Fisher’s LSD test.

Drought stress caused the higher accumulation of toxic MG, where maximum accumulation was under PEG-induced stress rather than water scarcity (Figure 10c). However, VA-pretreated seedlings showed a reduction in MG content by about 20 and 22% in the water shortage and PEG-induced stresses, respectively, compared to their respective stress alone. In addition, VA pretreatment in water scarcity stress brought the MG content to a maximum lower level, near the control condition.

In addition, Gly I activity was increased by 117 and 37% in W0- and PEG-treated seedlings, respectively, whereas Gly II activities were reduced by 63 and 46%, respectively, compared to the control. However, VA pretreatment further increased the activities of both Gly I and Gly II in tomato seedlings under both W0 and PEG treatment conditions in comparison with their respective stress alone (Figure 10a,b).

## 4. Discussion

Water deficit stress altered the water potential, and, therefore, plants suffered from water scarcity, which later caused other kinds of cellular dysfunction. Drought can adversely affect the biosynthesis of photosynthetic pigments and alter stomatal conductance, photosynthesis, and different enzymatic functions, which results in the inhibition of the plant’s growth and development [2]. However, various approaches have been implemented to attain higher plant tolerance upon water scarcity and are still under investigation. Against this backdrop, phenolic acid was used to prevent the drought-induced damage and growth retardation of tomatoes in this study. We made the artificial physical drought by withdrawing water, while 12% PEG was used to induce a physiological drought. Tomato growth was restricted by reducing the length of the shoot and root along with their biomass content. But a direct water deficit caused higher root growth retardation, while PEG-induced drought triggered a higher reduction in the shoot growth. It is likely that such drought-mediated plant growth reductions, such as FW and DW, were observed in previous studies [8,36,37]; therefore, it is very common in plants under water stress. It could be explained by an osmotic-stress-mediated alteration in the plant–water status, which is considered as the main reason for the reduction in cell division and expansion and restriction of the cell cycle transition [38,39]. The positive role of VA in increasing plant growth through increasing shoot and root lengths, along with their FW and DW under deficit water supply, was observed in the present study, and similar results were also reported from our previous study in salt-inundated tomato plants [17] and Cd-treated rice [16]. This VA-mediated growth improvement could be due to better tissue water, mineral uptake, photosynthesis, and enzymatic activity [39]. A drought-induced osmotic imbalance occurred due to the unavailability of water as well as a lack of optimum water in the root region, which later causes osmotic stress. Osmolyte accumulation like Pro can regulate the osmotic balance and protect plants from dehydration to some extent by increasing water uptake [17,39]. Drought-mediated higher Pro content was reported previously in tomatoes [39], *Cicer arietinum* [8], and *Zea mays* [40], which are similar to the findings of this study. Thereafter, further higher Pro accumulation in VA-pretreated seedlings disclosed a better osmotic status upon both water deficit and osmotic stress conditions, which was supported by elevated leaf RWC. This VA might be involved in regulating the Pro synthesis pathway for obtaining higher osmoregulation, which demands further research and is also corroborated by the report from An et al. [41]. With the support of other reports about phenolics like ellagic acid [42], caffeic acid [43], and gallic acid [44], which induce higher osmoregulation by elevating the biosynthesis of Pro in stressed plants, it could be confirmed that this group of metabolites are able to check the water content in stressed plants.

The drought resulted in the significant destruction of the Chl content along with a reduction in the intercellular CO_2_ concentration; the inhibition of Rubisco synthesis resulted in the loss of the photosynthetic rate as well as plant growth [45], which might be due to the alteration in enzymatic activities and excessive ROS generation [46]. However, in this study, VA pretreatment restored the reduction in Chl and Car content by lowering the leaf chlorosis in drought-stressed tomatoes. Such exogenous applications of different phenolic compounds, including apigenin, protocatechuic acid, and salicylic acid, contribute to improving chlorophyll formation as well as photosynthesis, thus resulting in better stress tolerance [19,39,47]. However, thorough details of phenolic-mediated mechanisms are still largely unexplored and demand further studies.

Drought stress causes the extreme generation of ROS like H_2_O_2_, mostly in the chloroplast and mitochondria of plant cells, due to the disruption in physiological events like photosynthesis and photorespiration. Eventually, plants suffer from oxidative stress, such as cellular damage, including EL, and lipid peroxidation, which is indicated by higher MDA content upon stress exposure [48,49]. Similar to Duc et al. [48] and Chitarra et al. [50], this study showed the seedlings suffering from drought-accumulated higher MDA and excessive EL, with an elevated level of H_2_O_2_ compared to the control as an indication of ROS-generated lipid peroxidation. However, drought-mediated H_2_O_2_ and MDA accumulations were lowered in VA-pretreated seedlings, which proved the protective role of VA in decreasing oxidative stress markers in tomato seedlings under water deficit. In our previous experiments, such VA-mediated lowering of ROS and lipid oxidation, along with better cell membrane stability, was observed in salt and Cd toxicity [16,17]. The supplemented VA induced such a decrease in H_2_O_2_, MDA, and EL in osmotically stressed plants, which might be due to the stronger involvement of ROS-scavenging antioxidant activities [41]. In addition, phenolic-induced plant tolerance against oxidative stress could be achieved through the active participation of the plant’s antioxidant defense mechanism to suppress ROS accumulation as well as reduce cellular damage [44,49,51].

Both enzymatic and non-enzymatic antioxidants work coordinately in a systematic way to scavenge excess ROS to keep its generation at a beneficial level in plant cells and thus contribute to the plant’s protection from oxidative damage [7]. In this well-organized mechanism, SOD shows its frontline defense by acting on the conversion of stress-induced O_2_^•−^ to H_2_O_2_, which is further detoxified by the activity of CAT [52]. Here, drought-treated seedlings showed elevated activities of both SOD and CAT as confirmation of extreme ROS generation. Therefore, VA pretreatment declined the activity of both SOD and CAT in drought-stressed seedlings, which can be described by the lower requirement of these enzymes’ activities due to a VA-mediated reduction in ROS. Non-enzymatic antioxidants of AsA and GSH scavenge H_2_O_2_ through the activities of APX, MDHAR, DHAR, and GR enzymatic components, as well as maintain the cellular redox balance of AsA:DHA and GSH:GSSG [7]. Here, the water deficit decreased the AsA content, with a higher level of its oxidized form of DHA, and increased the content of both GSH and GSSG in tomato seedlings. Such stress-induced reduction in AsA:DHA and GSH:GSSG with higher H_2_O_2_ generation specified the inadequacy of AsA and GSH for detoxifying this extreme H_2_O_2_. In opposition, VA pretreatment increased both AsA:DHA and GSH:GSSG by increasing AsA and decreasing the GSSG, along with lowering H_2_O_2_. Such a protective role of VA was described in our previous study on salt and metal toxicity [16,17]. In addition, an exogenous-protectant-mediated improvement in AsA:DHA and GSH:GSSG was observed in various stress-affected plants [53]. In addition, the nature of VA for increasing endogenous flavonoids and phenolic accumulation, which directly scavenge ROS in stressed plants, describes the VA-induced findings of the present study [19].

Seedlings showed elevated activities of APX, MDHAR, DHAR, and GR upon PEG-induced osmotic stress, while only DHAR activity was reduced with higher activity in the other three enzymes in water withdrawal situations, which follow the stressed-mediated higher H_2_O_2_ content. Previously, such a higher regulation of the enzymes’ activities of the AsA-GSH cycle was reported in various crops, including tomatoes, due to water deficit conditions [8,39]. However, VA pretreatment caused the lower activity of APX, MDHAR, DHAR, and GR in PEG-induced stressed seedlings, which were consistent with VA-mediated-less H_2_O_2_. Apart from DHAR activity, APX, MDHAR, and GR activities increased in stressed seedlings, which were, again, reduced in VA-pretreated conditions, which is consistent with H_2_O_2_ generation. Specifically, DHAR activity was increased in PEG-induced stress and reduced in water-withdrawal-induced stress conditions, but an alteration occurred in VA-pretreatment conditions. The reduction in APX, MDHAR, and DHAR activities, along with an incremental rise in AsA content in VA-pretreated seedlings upon stress conditions, described the lower need for such enzymes’ activities due to the VA-induced lower H_2_O_2_ generation. Our present results suggest that VA has a significant role in ROS scavenging by itself, which causes the lower requirement of AsA regeneration. As another significant component of the AsA-GSH cycle, GSH plays critical roles in H_2_O_2_ scavenging through GPX and/or GST activities [7,17]. The GSH also works in the process of AsA regeneration, which can be, again, used in H_2_O_2_ and also possesses xenobiotic properties, which detoxify xenobiotics along with the activity of GST [7]. In the present study, we found higher GSH content with elevated activity of GR, GPX, and GST in both kinds of drought stress. This might be the cause of a higher conversion of GSSG from GSH to obtain higher AsA for H_2_O_2_ detoxification, while GPX and GST caused the concurrent scavenging of extreme H_2_O_2_, which is consistent with previous findings [17,53]. On the converse, VA pretreatment allowed a significant reduction in GR, GPX, and GST activities, where the GSH and GSSG content were also lower in stressed seedlings, possibly due to the lower H_2_O_2_ generation. Such a VA-mediated alteration in the enzymatic antioxidants of the AsA-GSH cycle to regulate the AsA and GSH contents for scavenging extra H_2_O_2_ upon stress has been reported in the reports of Parvin et al. [17] in salt-stressed tomato plants and Bhuyan et al. [16] in Cd-stressed rice plants.

**Figure 11:**
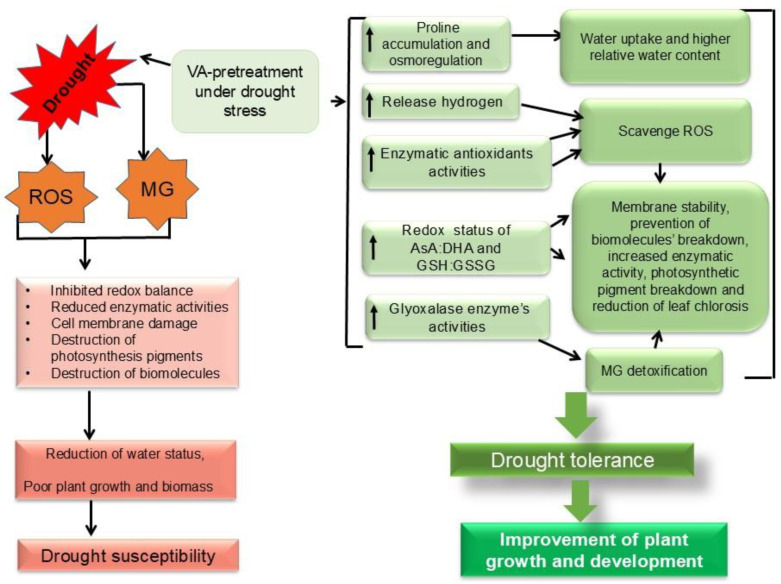
Possible mechanism of VA-induced enhancement of drought tolerance in tomato (ROS: reactive oxygen species; MG: methylglyoxal; VA: vanillic acid; AsA: ascorbic acid; DHA: dehydroascorbate; GSH: glutathione; GSSG: oxidized glutathione).

In addition, the glyoxalase system in the plant’s cell is stimulated to detoxify the elevated toxic MG content upon environmental stress, including drought [8]. The consequence of stress-mediated higher MG accumulation is cellular damage, including lipoprotein membranes. However, the plant can regulate this overproduced MG by the activities of Gly I, and Gly II, where GSH acts as a co-factor [54]. Our current study explored that instead of having higher Gly I activity, tomato seedlings suffered from acute MG toxicity with lowered Gly II activity upon drought conditions and thus showed an insufficient response from glyoxalase enzymes. Such findings are corroborated by the findings of other plant researchers who reported abiotic-stress-mediated higher MG generation [16,17,52,54]. The VA pretreatment caused the further upward activity of Gly I and Gly II in drought-stressed seedlings, resulting in reduced MG content, and thus confirmed the protective role of VA in the stimulation of glyoxalase enzymes. Similar results of VA-mediated abiotic stress tolerance through MG detoxification have been reported by Parvin et al. [17] and Bhuyan et al. [16] upon salt- and Cd-stressed plants, respectively. Consequently, a water-deficit-induced inhibition of tomato growth, osmotic stress, and photosynthetic pigments was associated with the excessive production of ROS. Thereafter, VA pretreatment improved the drought tolerance of tomato seedlings by regulating the AsA-GSH, along with different antioxidant enzymes and enzymes of the glyoxalase system. The mechanism involved in the VA-induced enhancement of drought tolerance related to the present study is summarized in Figure 11. As a mechanistic approach of VA, there was an augmentation in the endogenous proline, which acts as a significant osmotic regulator that can attenuate the drought stress. Thus, the VA-mediated elevation of Pro might be due to the higher involvement of Pro synthesis through upregulating the activity of both proline dehydrogenase (PDH) and pyrroline-5-carboxylate synthase (P5CS). Such VA-mediated higher Pro caused the betterment of osmotic status, which was confirmed by an increased relative leaf water content upon water shortage. Such VA-mediated higher Pro accumulation as a mechanistic approach for involving better drought tolerance was reported by An et al. [41] in blueberries. Upon a water shortage, ROS production becomes excessive, along with higher enzymatic activities like SOD, CAT, GPX, and GST, seems that the increased a activity of these enzymes were not enough to combat ROS toxicity. But VA reduced the activities of SOD, CAT, GPX, and GST enzymes, which disclosed the confirmation of VA-induced direct ROS scavenging due to the structural properties. In addition, VA-mediated a higher redox status of AsA:DHA and GSH:GSSG, along with higher levels of APX, MDHAR, and GR, which showed the possible involvement of a VA-induced AsA-GSH pool to reduced oxidative stress upon a drought, which could be related to a lower level of H_2_O_2_ in VA-pretreated conditions. Such upregulation of antioxidants through VA supplementation in drought stress can be correlated with a previous finding [41], where the enhanced expression levels of antioxidant enzyme genes (*Fe-SOD*, *Cyt Cu/Zn-SOD*, *Chl Cu/ZnSOD*, *CAT*, *GPX*, *GSH-Px*, *APX*, *DHAR*, *MDHAR*, and *GR*), increased the AsA and GSH contents. Finally, the VA-mediated restoration of chlorophyll content like Chl *a*, Chl *b*, and Car upon drought showed the possible involvement of VA in the chlorophyll synthesis pathway and the regulation of chlorophyllase enzymatic activity. From our point of view, there is the VA-mediated upregulation of antioxidants, and the glyoxalase system and osmoregulation are the main mechanistic approaches to enhance drought tolerance by enhancing the tomato seedling growth and chlorophyll contents.

This regulatory role of VA from this study showed us the hope of drought management in field conditions, which demand further detailed field trials (with different durations of drought, different doses of VA, and various application methods), focusing on the yield response along with the growth parameters.

## 5. Conclusions

The protective role of VA significantly improves the tomato’s tolerance to water-deficit-induced oxidative damage. Drought stress caused the inhibition in the seedling growth, development, and photosynthesis process; conversely, VA-pretreated seedlings showed a better recovery from stress-induced growth inhibitions. Plants suffering from drought stress-mediated extreme ROS generation and consequent oxidative damage weredecreased through VA application. Hence, VA restricted the drought-induced ROS, which can be explained by the responses in antioxidant activities, while the VA-mediated lower MG generation was due to the higher activity of glyoxalase enzymes. In addition, a better osmotic balance, photosynthetic pigment accumulation, and finally, higher seedling growth in VA-pretreated seedlings upon stress conditions support the VA-mediated improvement in tomato tolerance to drought. Moreover, the role of VA in improving tomato tolerance against drought stress is clear from these results. Furthermore, comprehensive experiments are required to be conducted in the laboratory and in-field conditions to evaluate the signaling roles of VA and the effects of VA on other physiological processes and yield-improving capacities.

## Data Availability

All data are available in this article.
